# Stable Epigenetic Variants Selected from an Induced Hypomethylated *Fragaria vesca* Population

**DOI:** 10.3389/fpls.2016.01768

**Published:** 2016-11-29

**Authors:** Jihua Xu, Karen K. Tanino, Stephen J. Robinson

**Affiliations:** ^1^Department of Plant Sciences, University of SaskatchewanSaskatoon, SK, Canada; ^2^Agriculture and Agri-Food Canada, Saskatoon Research CentreSaskatoon, SK, Canada

**Keywords:** DNA methylation, *Fragaria vesca*, epigenetics, selection, early flowering

## Abstract

Epigenetic inheritance was transmitted through selection over five generations of extreme early, but not late flowering time phenotypic lines in *Fragaria vesca*. Epigenetic variation was initially artificially induced using the DNA demethylation reagent 5-azacytidine (5-azaC). It is the first report to explore epigenetic variant selection and phenotypic trait inheritance in strawberry. Transmission frequency of these traits was determined across generations. The early flowering (EF4) and late stolon (LS) phenotypic traits were successfully transmitted across five and three generations through meiosis, respectively. Stable mitotic transmission of the early flowering phenotype was also demonstrated using clonal daughters derived from the 4th Generation (S4) mother plant. In order to further explore the DNA methylation patterns underlying the early flowering trait, the standard MSAP method using isoschizomers Hpa II/Msp I, and newly modified MSAP method using isoschizomers Tfi I/Pfe I which detected DNA methylation at CG, CHG, CHH sites were used in two early flowering lines, EF lines 1 (P2) and EF lines 2 (P3), and control lines (P1). A significant reduction in the number of fully-methylated bands was detected in P2 and P3 when compared to P1 using the novel MSAP method. In the standard MSAP, the symmetric CG and CHG methylation was maintained over generations in the early flowering lines based on the clustering in P2 and P3, the novel MSAP approach revealed the asymmetric CHH methylation pattern was not maintained over generations. This study provides evidence of stable selection of phenotypic traits, particularly early flowering through both meiosis and mitosis, which is meaningful to both breeding programs and commercial horticulture. The maintenance in CG and CHG methylation over generations suggests the early flowering phenotype might be related to DNA methylation alterations at the CG or CHG sites. Finally, this work provides a new approach for studying the role of epigenetics on complex quantitative trait improvement in strawberry, as well as providing a tool to expand phenotypic diversity and expedite potential new horticulture cultivar releases through either seed or vegetative propagation.

## Introduction

Epigenetic marks can be passed down from parent to offspring without altering the primary DNA sequence. DNA methylation, histone modification, and small RNA interference are all epigenetic marks. DNA methylation patterns are the most heritable marks and can be associated with patterns of histone modification, regulating gene transcription (Feng and Jacobsen, 2011). Additionally, small RNAs are also involved in the induction of DNA methylation (RNA directed DNA methylation) and histone modification processes (Law and Jacobsen, 2010). The composition of these marks together affect the conformation, topology of chromatin, regulation of gene expression, controlling growth, and developmental transitions in plants (Hirsch et al., 2012; Verkest et al., 2015). When these modifications interact with the protein coding genes in the genome, alterations to phenotypic traits can be generated (Becker and Weigel, 2012). These phenotypic characteristics may be of significant commercial value but incorporation of valuable traits through traditional breeding approaches takes considerable time. Horticulture fruit crops are clonally propagated and the strategy to induce and fix a phenotype has a relatively high potential for successful release of new cultivars. We used the DNA demethylation reagent 5-azacytidine (5-azaC) as a tool to induce and broaden phenotypic diversity in *Fragaria vesca*. In a previous study, we showed 5-azaC does not induce mutations to the primary DNA sequence (Xu et al., 2016).

DNA methylation as the most well-studied epigenetic mark, occurs at the symmetrical methylated sites CG and CHG (H = A, C, T) in the DNA context, and non-symmetrical cytosine (CHH) in plants (Meyer et al., 1994). The methylation sensitive restriction enzymes are normally used to screen DNA methylation polymorphism in a large number of lines in a cost-effective and efficient way. Isoschizomers are enzymes having the same recognition site except one enzyme is methylation sensitive and the other is methylation insensitive (Bird and Southern, 1978). In the standard MSAP method, the isoschizomers *Hp*a II and *Msp* I are often exploited as frequent cutters to replace *Mse* I in AFLP but detect genome-wide DNA methylation (Cedar et al., 1979; Vos et al., 1995; Reyna-López et al., 1997). Due to the efficiency and low cost, numerous studies have used this method to determine cytosine methylation in different fields from tissue culture (Chakrabarty et al., 2003; Xu et al., 2004), environmental stress (Shan et al., 2013; Rico et al., 2014), to ecology (Bossdorf et al., 2010; Schulz et al., 2013), and 5-azaC treated plant material (Sano et al., 1990; Fieldes, 1994; Marfil et al., 2012). However, since the recognition site of *Hp*a II and *Msp* I is 5′-CCGG-3′ (CG and CHG contexts), this method can only investigate DNA methylation based on 5′-CCGG-3′. New isoschizomers *Tfi* I and *Pfe* I can recognize 5′-GAWTC-3′ (CG, CHG, and CHH contexts). Therefore, in order to study the DNA methylation profiles at all CG, CHG, and CHH methylation contexts, standard MSAP using *Hp*a II/*Msp* I together with a new MSAP using *Tfi* I/*Pfe* I were applied in our study. Using a suite of isoschizomers provides a more comprehensive tool to help identify the epialleles controlling phenotypic traits of interest.

Studies related to the inheritance and selection of phenotypic traits induced by epigenetic variations have been reported in the past two decades. In *Arabidopsis*, continuous distribution and variation was observed in complex quantitative traits such as flowering time, plant height and plant growth in epigenetic Recombinant Inbred Lines (epiRILs) having a similar genome but varying DNA methylation levels (Johannes et al., 2009; Reinders et al., 2009). DNA methylation status was further demonstrated to be transmitted from parent to offspring through both mitosis and meiosis in *Arabidopsis* (Kakutani et al., 1999). The mitotic inheritance is referred to as the propagation of epigenetic modifications through somatic cell division (Martin and Zhang, 2007). Transgenerational meiotic inheritance of quantitative traits such as flowering time and plant height were also reported in families of epiRILs in *Arabidopsis* (Johannes et al., 2009; Zhang et al., 2013). In *Brassica*, the transgenerational inheritance of seed size, plant stature and floral morphology has been examined after 5-azaC treatment of seeds (Amoah et al., 2012).

Not all epigenetic marks are transgenerationally heritable, with many being reset at meiosis which likely causes the subsequent disappearance of phenotypic traits induced by epigenetic changes in the following generations (Hauben et al., 2009; Falke et al., 2013). In the *Arabidopsis* epiRILs study, after two to five generations, DNA remethylation occurred in some of the hypomethylated variants (Johannes et al., 2009). In *Brassica napus* lines, the acquired epigenetic component disappeared in the progenies and the phenotypic trait of lower respiration rate returned to the level of control (Hauben et al., 2009). In the stress-induced *Arabidopsis* lines possessing epigenetic memory, the modified epigenome can be transmitted to the next generation, but it disappeared after two generations without stress treatment (Suter and Widmer, 2013).

Nevertheless, inherited epialleles are possible under selection even though these epigenetic patterns may be transient. Underlying the transgenerational high heritability in flowering time and plant height, stable epialleles across the genome play an important role in this process (Johannes et al., 2008, 2009). The generation of epiallelic variation and the resultant phenotypic diversity in seed size and composition that allowed selection was demonstrated in *Brassica rapa* inbred lines (Amoah et al., 2012). In *Arabidopsis*, a quantitative genetics approach examined the heritability of flowering time, plant height, total biomass, and root:shoot ratio in different environmental conditions which showed the action of applied selection on phenotypic plasticity (Zhang et al., 2013). The artificial selection of extreme lines which might contain the phenotypic-related epialleles were reported in *B. napus*, the selected lines were self-fertilized and families were generated and evaluated for energy use efficiency (EUE). Following rounds of recurrent selection, higher EUE was successfully inherited through the generations and were distinguished based on epigenetic (DNA methylation and histone modification) status (Hauben et al., 2009). Therefore, recurrent selection for certain specific phenotypic traits associated with epialleles such as EUE may enable breeders to fix those traits in crops of commercial value.

The Rosaceae family members such as strawberry (*F. vesca, F*. × *ananassa*), are commercially propagated through mitotic generations (clonal “daughters”) as well as meiotically (achenes, seeds) for breeding purposes. However, selection of stable epigenetic mutants in strawberry or other Rosaceae family members has not yet been investigated. The Rosaceae family contains the most important horticulture temperature fruit crops including apples, cherries, peaches, pears, plums as well as ornamental plants such as roses. *F. vesca* is a useful model system to examine the transmission of phenotypic traits induced by epigenetic variation due to its short 3.5 month seed to seed life cycle in the greenhouse and modified stems (stolons) to clonally propagate daughter plants. Here, we used the diploid *F. vesca* lines identified from the hypomethylated population generated in a previous study (Xu et al., 2016) to investigate the inheritance of variant traits including early and late flowering time, and late stolon emergence time. The question of whether the lines with specific phenotypic traits possess varied epigenetic status and can be subject to selection was explored in this study. Since *F. vesca* can propagate either through generative or vegetative processes, it is an interesting model system to test the transmission of flowering time through both meiosis and mitosis from the same mother plants. This is the first report to explore epigenetic variants selection and phenotypic trait inheritance in strawberry and presents a new approach to accelerate breeding by inducing heritable epigenetic lines of horticultural importance through 5-azaC application.

## Materials and methods

### Plant materials

The plant materials examined in this research represent successive generations of control and hypomethylated families of *F. vesca* ssp. *vesca* accession Hawaii 4 eighth generation of inbreeding (H4S8). The hypomethylated population of H4S8 was produced by treating seeds with the DNA demethylating reagent 5-azacytidine (5-azaC) (Xu et al., 2016). This original generation (H4S8) is referred to as S0. Fifty-nine lines represented control (no 5-azaC treatment) and the remaining 305 lines were treated with 5-azaC. These lines are a direct descendent of the plant (H4S4) *F. vesca* on which the reference genome was sequenced (Shulaev et al., 2011). We confirmed that the generation S0 is genetically identical but epigenetically unrelated since plants possess expanded variation in DNA methylation caused by a loss of methylation at random loci at the sites of 5-azaC incorporation, following a dose-response function (Xu et al., 2016). This additional epigenetic variation results in expanded phenotypic variation following a hidden stochastic function. The hypomethylated individuals in subsequent generations (S1 through S5) are related, as families derived from selfing.

To follow the pedigree of the plant material throughout the experiment, all the 5-azaC treated lines and control lines were assigned a sub-code to the original names provided to each line (ERFv1–ERFv364). All *F. vesca* plants grown in this study followed the same procedure: dried achenes (seeds) were placed on wet filter paper in petri dishes and allowed to imbibe and germinate before being transplanted to soil and grown in the greenhouse at 23 ± 2°C day and 18 ± 2°C night with 18/6 h day/night photoperiod.

Pollination bags were placed over opening strawberry flowers for 7 days to ensure the next generation of achenes was derived from self-pollination. The achenes were harvested from mature berries, dried under low heat using a food dehydrator before being harvested and maintained separately. The S-suffix number denotes each successive generation of selfing, where the generations S0 through S5 represent the inbred generations H4S8 through H4S13.

### Phenotypic assessment of quantitative traits

The scoring of flowering time was recorded as the number of days from sowing to anthesis, determined by the opening of the first (primary) flower; stolon emergence time was scored as the number of days from sowing to the time when the first stolon was observed on the mother plant. To account for differences observed among repeated measurements conducted across time and seasons, a control population was grown at each generation and all plants were randomized on the greenhouse bench.

### Artificial selection for the desired traits

**Figure 1:**
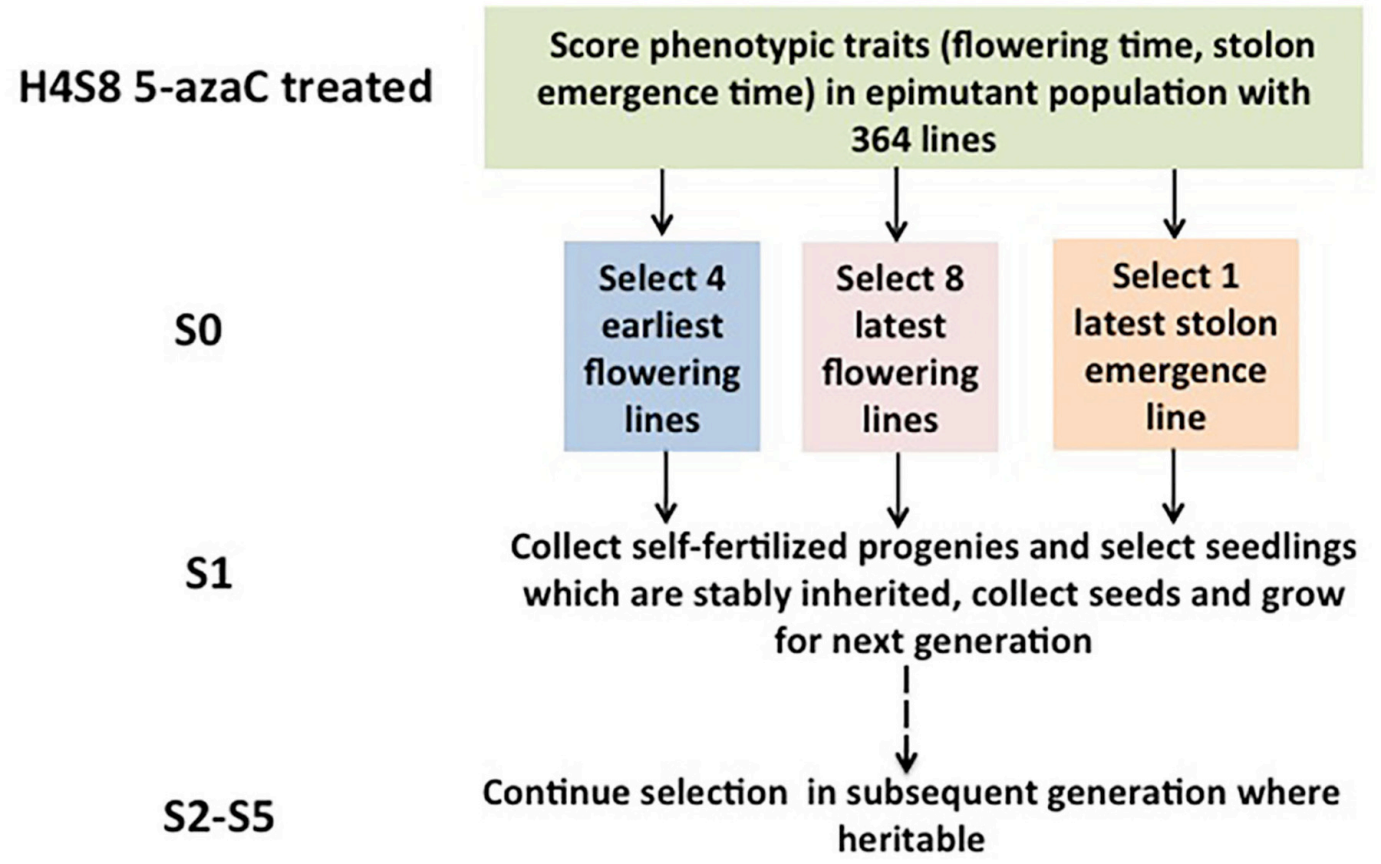
**Selection for quantitative traits across generations in *Fragaria vesca***. The selection was started with the hypomethylated seedling population with a genetically identical background derived from 5-azacytidine treated seeds.

A strict pedigree method was used based on families, and a summary of the number of lines assayed per family is presented (Figure 1). Seeds were collected from the selected S0 plants and were grown to maturity to produce the subsequent generation 1 (S1). The phenotypic traits of the S1 population were recorded. The most extreme individuals within the S1 generation were selected to produce generation 2 (S2), rather than the family means. The selection based on the individuals within the most extreme family was used in S2, and the next rounds of selection in the subsequent generations followed the same rule. A total of five generations (S1, S2, S3, S4, S5) were selected for early flowering time, three generations (S1, S2, S3) were selected for late stolon emergence time, and two generations (S1, S2) were selected for late flowering time.

**Figure 2:**
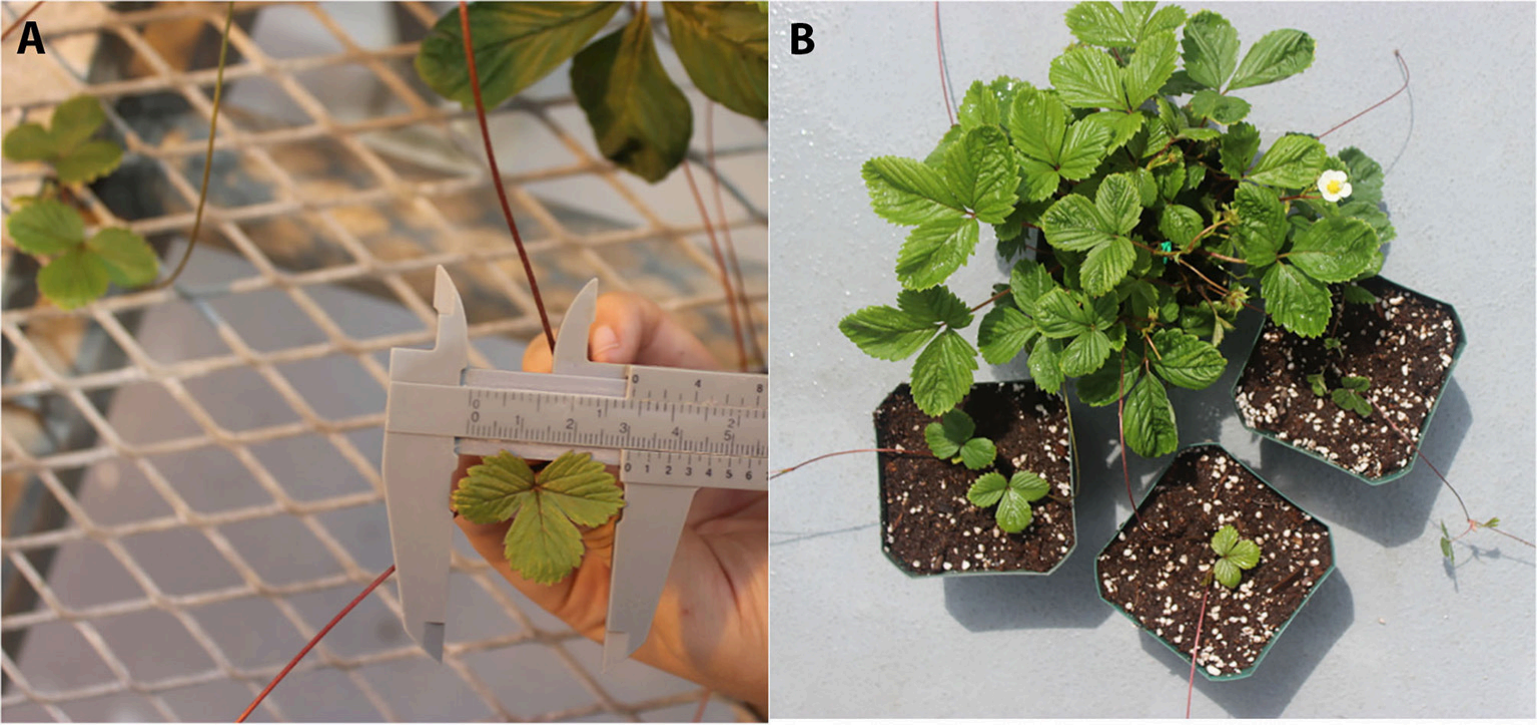
**Example of clonal daughter plants derived from vegetative stolons in *Fragaria vesca*. (A)** The first triple leaves (size 3 cm) from the primary daughter plant; **(B)** Three daughter plants from the same mother plant were transferred into new pots.

In the evaluation of transgenerational inheritance of flowering time through mitosis, three daughter plants of the S5 generation derived from the first three stolons of each of five mothers studied were transferred into new four-inch pots when the width of the first triple leaves was 3 cm as shown in Figure 2. A total of 15 plants (5 × three daughter plants) were derived from each of three S4 generation families (EF4-9-13-15, EF4-14-7-6, EF4-14-7-14) with the average flowering time of three daughter plants (three replications) used for data analysis. Flowering time was scored as the number of days from transplanting daughter plants to anthesis determined by the opening of the primary flower of the inflorescence.

**Figure 3:**
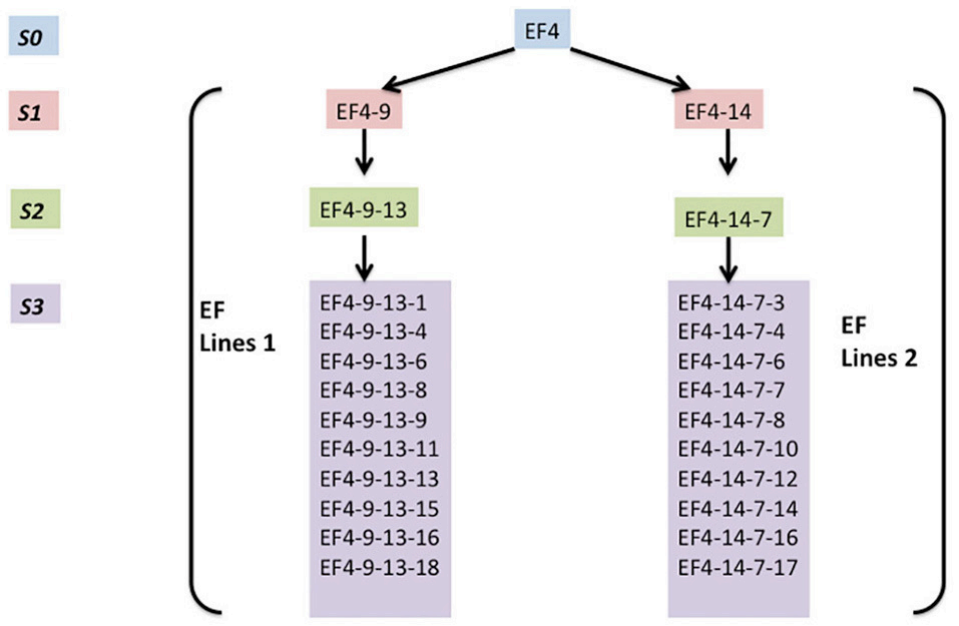
**The overview of two early flowering lines (EF4 lines 1, EF4 lines 2) composed of four generations**. EF4 is an early flowering line selected from a hypomethylated population treated with 5-azaC.

H4S8 lines and two early flowering lines EF lines 1 and EF lines 2 (Figure 3) were used in the MSAP experiment. These were collected from different generations (S1, S2, and S3) of progeny from the early flowering line EF4 (ERFv153) in the hypomethylated population treated with 5-azaC. EF4 did not possess genetic polymorphism to its control population in assays for SNPs using short read based whole genome sequencing (Xu et al., 2016).

The novel MSAP protocol using *Tfi* I/*Pfe* I with *Bgl* II was similar to the MSAP protocol described using *Hpa* II/*Msp* I with *Eco*R I (Xu et al., 2016) except the enzymes and corresponding NEB buffer were replaced. The adaptors and primers used in these standard and novel MSAP are listed in Tables 1, 2. The amplified MSAP products were resolved using exactly the same method as the MSAP protocol previously described in Xu et al. (2016).

**Table 1:** **Sequences of adaptors and primers used for pre-selective amplification and selective amplification in standard MSAP method using *Eco*R I and isoschizomers *Hpa* II/*Msp* I**.

**Adapters/Primers**	***Eco*R I**	***Hpa* II/*Msp* I**	
Adapter	5′-CTCGTAGACTGCGTACC-3′	5′-GACGATGAGTCTAGAA-3′	
	3′-CATCTGACGCATGGTTAA-5′	3′-CTACTCAGATCTTGC-5′	
Pre-amplification primers	5′-GACTGCGTACCAATTC A-3′	5′-GATGAGTCTAGAACGG T-3′	
Selective primers	5′-GACTGCGTACCAATTC ACT-3′	5′-GATGAGTCTAGAACGG TAA-3′	
	5′-GACTGCGTACCAATTC ACT-3′	5′-GATGAGTCTAGAACGG TTG-3′	
	5′-GACTGCGTACCAATTC ACA-3′	5′-GATGAGTCTAGAACGG TTA-3′	
	5′-GACTGCGTACCAATTC ACA-3′	5′-GATGAGTCTAGAACGG TTT-3′	

**Table 2:** **Sequences of adaptors and primers used for pre-selective amplification and selective amplification in new MSAP method using *Bgl* II and isoschizomers *Tfi* I/*Pfe* I**.

**Adapters/Primers**	***Bgl* II**	***Tfi* I/*Pfe* I**
Adapter	5′-CTCGTAGACTGCGTACC-3′	5′-GACGATGAGTCTAGAA-3′
	3′-CATCTGACGCATGGCTAG-5′	3′-CTACTCAGATCTTTWA-5′
Pre-amplification primers	5′-GACTGCGTACCGATCT A-3′	5′-GATGAGTCTAGAAAWTC C-3′
Selective primers	5′-GACTGCGTACCGATCT AGT-3′	5′-GATGAGTCTAGAAAWTC CAT-3′
	5′-GACTGCGTACCAATTC AGT-3′	5′-GATGAGTCTAGAAAWTC CTT-3′
	5′-GACTGCGTACCAATTC AGT-3′	5′-GATGAGTCTAGAAAWTC CCT-3′
	5′-GACTGCGTACCAATTC AGA-3′	5′-GATGAGTCTAGAAAWTC CAT-3′
	5′-GACTGCGTACCAATTC AGA-3′	5′-GATGAGTCTAGAAAWTC CTT-3′
	5′-GACTGCGTACCAATTC AGA-3′	5′-GATGAGTCTAGAAAWTC CCT-3′

### Statistical analysis

Phenotypic measurements made in the hypomethylated S0 generation were ranked to identify the lines expressing the extreme phenotypes. The mean and variance of each trait measured in the control S0 generation was determined by the *Z*-test to evaluate if each individual was significantly different from the control mean. In the following generations, they were subjected to statistical analysis using two-sample Student's *t*-test. Statistical analysis on the data of each trait measured used a 95% confidence interval to select individuals as potentially belonging to a population that differs from the control population. Comparisons between hypomethylated lines and control lines were restricted to those grown at the same time. All data was analyzed using statistical software R.

In order to establish a standard to compare the proportion of early and late flowering lines in the epimutant and control population, we defined the lines with flowering time less than the mean of control minus one standard deviation (*SD*; mean − *SD*) as early flowering lines, and similarly the lines with flowering time greater than the control plus one standard deviation (mean + *SD*) as late flowering lines. A similar process was applied to the late stolon emergence lines. We defined the lines with stolon emergence time greater than the control plus one standard deviation (mean + *SD*) as late stolon emergence time lines. The equality of proportions of early flowering, late flowering, and late stolon emergence time in epimutant and control lines in each generation was evaluated by Chi-square. MSAP profile analysis was determined using Principal Coordinate Analysis (PCoA) and Analysis of Molecular Variance (AMOVA) as described (Xu et al., 2016).

## Results

### Initial selection of lines with extreme phenotypic traits (S0 and S1 generations)

**Figure 4:**
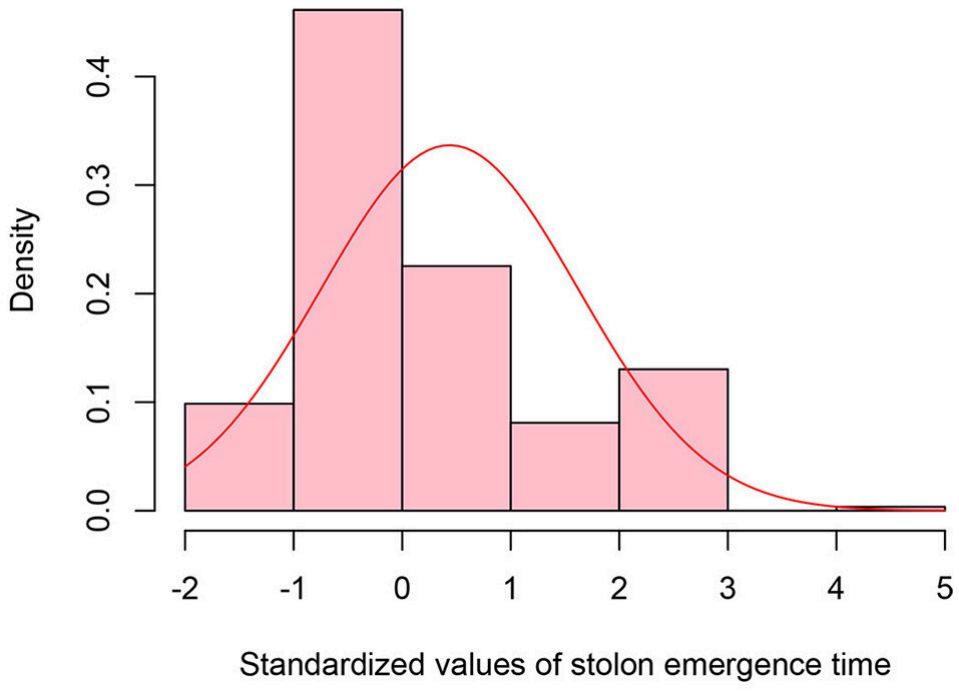
**Distribution of standardized values for stolon emergence time observed in the epimutangenized population relative to control population mean**. Stolon emergence time density histogram of *Z*-test values from 284 hypomethylated lines.

The S0 generation was used to initially select extreme phenotypes of early and late flowering and late stolon emergence. In total, four early flowering lines (ERFv148, ERFv157, ERFv168, ERFv153, renamed EF1–EF4, respectively), eight late flowering lines (ERFv16, ERFv132, ERFv127, ERFv131, ERFv134, ERFv140, ERFv141, ERFv138, renamed LF1–LF8, respectively; Xu et al., 2016), and a single line expressing late stolon (LS) development (Figure 4) were selected as possessing the most extreme phenotype that significantly differed from the control population.

**Figure 5:**
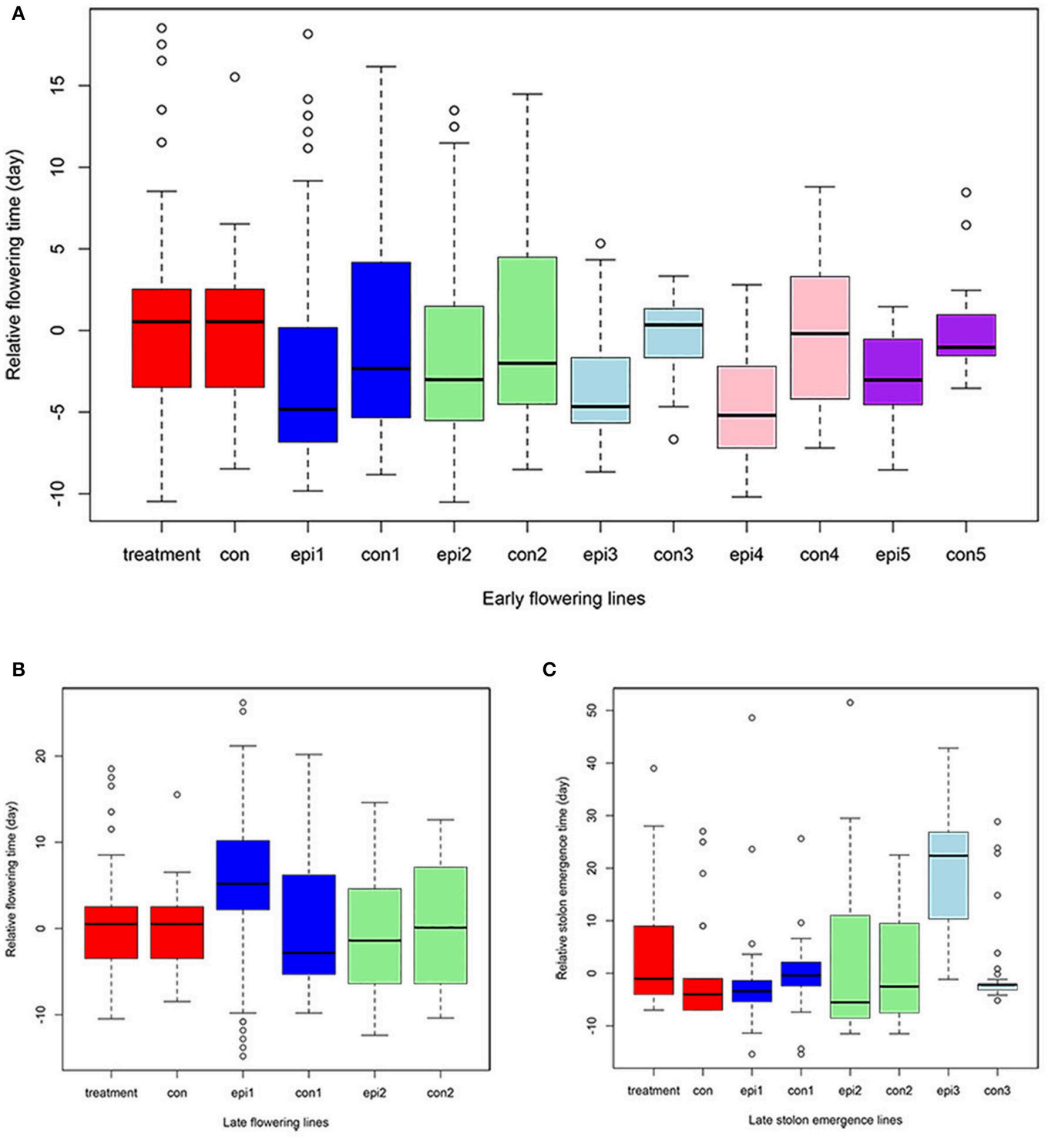
**Quantitative traits distribution across generations**. The Y axis represents the relative value compared to the corresponding control (con) in early flowering time **(A)**, late flowering time **(B)**, and stolon emergence time **(C)** phenotypes. X axis, treatment and con are from the S0 generation epimutant lines and control lines; epi1 and con1 represent the S1 epimutant progenies and control lines; epi2 and con2 are S2 epimutant progenies and control lines; epi3 and con3 are S3 epimutant progenies and control lines; epi4 and con4 are S4 epimutant progenies and control lines; epi5 and con5 are S5 epimutant progenies and control lines.

ERFv153 (EF4) was selected for DNA methylation and DNA sequencing analysis, in which the greatest changes in DNA methylation level but no primary DNA sequence changes in genes controlling flowering time were detected (Xu et al., 2016). The early flowering sample sizes in each family of the S1 generation varied. The smallest population comprised only seven individuals and the largest consisted of 22 individuals, while the control family was composed of 36 individuals (Table 3). This variation in family size was due to a combination of seed availability or plant survival after transplantation. The mean and standard deviation for flowering time distribution of the control population in the S1 generation was 65.8 and 6.9 days, respectively. By contrast, the flowering time mean values for the families derived from hypomethylated lines ranged from 60.6 to 66.6 days with standard deviations ranging from 5.6 to 8.3 days (Table 3). The greatest significant difference was observed between the control and EF1 families where a difference of 5 days separated the means. Although no difference was detected in the proportion of early flowering and control lines in families of the S0 generation, a significant difference in the S1 generation was detected (*p* = 0.03; Table 4). In the S1 generation, the percentage of early flowering individuals (33.3%) in the early flowering epimutant lines was more than twice the percentage (13.9%) of control lines. When all families in the S1 generation based on phenotypic traits were combined (Figure 5A), the proportion of early flowering individuals was higher in the early flowering epimutant lines than in control lines.

**Table 3:** **Phenotypic trait properties of selected lines across different generations**.

**Phenotypic trait**	**S1 generation**				**S2 generation**				**S3 generation**				**S4 generation**			
	**Families**	**Mean**	***SD***	**Size**	**Families**	**Mean**	***SD***	**Size**	**Families**	**Mean**	***SD***	**Size**	**Families**	**Mean**	***SD***	**Size**
Early flowering	EF1^*^	60.6	5.6	21	EF1-3	75.3	3.8	12	EF4-9-6^*^	68.7	3.5	20	EF4-9-13-15^*^	67.8	3.3	22
	EF2	66.5	5.9	22	EF1-5	78.5	5.6	15	EF4-9-10^*^	69.8	3.1	18	EF4-14-7-6^*^	69.4	3.1	27
	EF3	66.6	8.3	7	EF1-10	79.5	6.9	22	EF4-9-13^*^	69.2	1.7	18	EF4-14-7-14^*^	68.6	2.9	30
	EF4	62.9	7.2	19	EF1-20	79.8	7.3	19	EF4-14-3^*^	69.8	3.9	18	Control	73.2	4.7	20
	Control	65.8	6.9	36	EF4-9^*^	71.6	2.5	16	EF4-14-7^*^	67.8	1.9	20				
					EF4-14^*^	72.3	2.7	17	Control	72.7	2.2	45				
					EF4-18	75.8	2.8	21								
					Control	77.5	5.9	60								
Late flowering	LF1^*^	88.2	9.6	19	LF7-3	79.5	7	15								
	LF2	73.1	8.4	18	LF7-4	80.5	7.1	8								
	LF3	80.3	4	12	LF7-6	78.4	6.6	30								
	LF4	81.9	2.3	11	LF7-7	79	6.7	8								
	LF5^*^	83.6	2.3	26	LF8-1	76.8	9.8	4								
	LF6^*^	87.9	9.7	7	LF8-5	78.8	6.7	11								
	LF7^*^	91.7	6.5	14	LF8-7	79.5	6.3	23								
	LF8^*^	86.7	3	7	Control	79.4	7.1	28								
	Control	76.5	7.1	37												
Late stolon emergence	LS	59.1	8.6	55	LS-54	56.1	9.9	32	LS-56-10^*^	61	12.3	24				
	Control	61.4	5.6	55	LS-56^*^	65.5	17.6	28	LS-56-25^*^	69	8.6	28				
					Control	58.5	10.3	32	Control	45.2	7.5	45				

* *p < 0.05 “Size” = the number of individuals*.

**Table 4:** **Composition of the early flowering lines in each generation in epimutant and control lines**.

**Generation**	**Early flowering epimutant**			**Control**			**χ^2^**	***p*-Value**
	**Number of early flowering lines**	**Number of lines**	**%**	**Number of early flowering lines**	**Number of lines**	**%**		
S0	65	293	22.2	14	59	23.7	0.07	0.80
S1	23	69	33.3	5	36	13.9	4.57	0.03
S2	14	61	22.9	4	30	13.6	1.17	0.28
S3	70	94	74.5	6	45	13.3	45.90	<0.001
S4	43	79	54.4	4	20	20	7.59	0.01
S5	18	36	50	1	24	4.2	13.98	<0.001

*Early flowering lines were determined by the number of lines with flowering time less than the mean of the control minus one standard deviation (mean − SD)*.

The transmission of the late flowering trait focused on eight families with population sizes ranging between 7 and 26, these were compared to a control family consisting of 59 individuals (Table 3). The control family mean was 76.5 days to flowering with a standard deviation of 7.1 days. The mean and standard deviation values describing the distributions of the hypomethylated families ranged from 73.1 ± 8.4 to 91.7 ± 6.5 days. The greatest difference between control and hypomethylated family means was around 14 days. Similar to the observation made on the early flower lines, when all hypomethylated lines were combined, there were individuals at the extremes of the entire distribution from families LF7 and LF8 where their flowering time family mean also was significantly late compared to the control mean value. Similar to early flowering lines, although no difference was detected in the proportion of late flowering and control lines in families of the S0 generation, a significant difference (*p* = 0.01) of late flowering individuals in the late flowering epimutant lines was also observed when compared to control lines in the S1 generation, with the percentage of late flowering lines more than twice the control in the S1 generation (Table 5). When all families in the S1 generation based on phenotypic traits were combined (Figure 5B), the proportion of late flowering individuals was higher in the late flowering epimutant lines than in control lines.

**Table 5:** **Composition of the late flowering lines in each generation in epimutant and control lines**.

**Generation**	**Late flowering epimutant**			**Control**			**χ^2^**	***p*-value**
	**Number of late flowering lines**	**Number of lines**	**%**	**Number of late flowering lines**	**Number of lines**	**%**		
S0	33	293	11.3	10	59	17	1.48	0.22
S1	38	107	35.5	9	59	15.3	7.69	0.01
S2	18	99	18.2	7	28	25	0.64	0.42

*Late flowering lines were determined by the number of lines with flowering time greater than the mean of the control plus one standard deviation (mean + SD)*.

A total of 38 out of 285 lines showed significantly late stolon emergence time (Figure 4). One extremely late stolon emergence line named LS was selected to study the transmission of stolon emergence time. The phenotypic assessment for stolon emergence revealed data that are positively skewed, differing significantly from a normal distribution. In the S0 generation, the percentage of late stolon emergence individuals (21.6%) in the epimutant lines was twice the percentage of control lines (10%), similar to the flowering time trait in the S0 generation, no significant difference was detected (Table 6). Whereas in the S1 generation, this percentage was decreased to the level of about one-third (3.6%) of control lines (10.9%; Figure 5C, Table 6). However, two extremely late stolon emergence lines (LS-54, LS-56) were found in the S1 generation (Table 3). These two individuals were selected and self-fertilized to generate the S2 generation.

**Table 6:** **Composition of the late stolon emergence lines in each generation in epimutant and control lines**.

**Generation**	**Late stolon emergence epimutant**			**Control**			**χ2**	***p*-value**
	**Number of late stolon emergence lines**	**Number of lines**	**%**	**Number of late stolon emergence lines**	**Number of lines**	**%**		
S0	61	283	21.6	5	50	10	3.57	0.06
S1	2	55	3.6	6	55	10.9	2.16	0.14
S2	15	60	25	8	32	25	0.00	1.00
S3	42	52	80.8	4	45	8.9	49.99	<0.001

*Late stolon emergence lines were determined by the number of lines with stolon emergence time greater than the mean of the control plus one standard deviation (mean + SD)*.

These initial S0 and S1 results provide evidence of the effect of selection and the inheritance of flowering time through meiosis. The priority was given to the most extreme individuals in the family regardless of the mean difference compared to control. Therefore, two early flowering lines EF1, EF4 and two late flowering lines LF7 and LF8 were retained as they contained the earliest and latest flowering individuals. In total, three or four individuals with extremely early or late flowering time from each of these four families were selected and self-fertilized to produce the S2 generation (Table 3).

### The transmission of flowering time and stolon emergence time in selected lines from S2 to S3 generations

In the seven S2 generation families identified with the early flowering trait, there were two families, EF4-9 and EF4-14, which had significantly earlier flowering time (Table 3). It was observed that 22.9% contained early flowering individuals, with only 13.6% of early flowering individuals in the control (Table 4). No significant difference was observed between the two combined early flowering families (EF4-9 and EF4-14) and control (*p* = 0.28). Since the early flowering trait was largely contributed by the families of EF4-9 and EF4-14, the five individuals with the most extreme early flowering phenotypes EF4-9-6, EF4-9-10, EF4-9-13, EF4-14-3, and EF4-14-7 were selected from these two families for study in future generations (Table 3).

By contrast, in the seven S2 generation families selected for the late flowering trait, all failed to transmit the late flowering phenotype (Table 3, Figure 5B). There was no significant difference detected in the S2 generation between the late flowering and control families (Table 5). Thus, the late flowering phenotype was discontinued in the selection process.

In the two late stolon emergence families selected from the S2 generation, the family LS-56 had significantly late stolon emergence time whereas the family LS-54 had no significant difference in emergence time (Table 3). When both S2 generation families LS-54 and LS-56 were combined, the percentage of late stolon emergence individuals were equivalent to the control lines (25.0%, Table 6). Furthermore, no significant difference was observed between the late stolon emergence families and control (Table 6). Nevertheless, two extremely late stolon emergence individuals (LS-56-10, LS-56-25) from the family LS-56 were observed, and these two individuals were selected for the subsequent study of the transmission of late stolon emergence time to S3.

By the S3 generation, the families of all five selected early flowering lines (EF4-9-6, EF4-9-10, EF4-9-13, EF4-14-3, and EF4-14-7) had significantly earlier flowering time when compared to the control (Table 3). When combining all five families selected, the percentage of early flowering individuals in the early flowering epimutant lines (74.5%) was nearly six times the percentage of control lines (13.3%, Table 4) and a highly significant difference (*p* < 0.001) was detected between early flowering families and the control. Three of the earliest flowering individuals (EF4-9-13-15, EF4-14-7-6, EF4-14-7-14) were selected to evaluate transmission of this trait to one more generation. Similarly, the progenies of two selected late solon emergence families (LS-56-10, LS-56-25) also indicated significantly late stolon emergence time (Table 3). The percentage of late stolon individuals in the late stolon emergence epimutant lines (80.8%) was also highly significant (*p* < 0.001) at nine times the percentage of control lines (Table 6).

### The transmission of early flowering time from S4 to S5 through meiosis and mitosis

The transmission of the early flowering time phenotype was continued in the subsequent S4 and S5 generations. Consistently, all S4 generation families derived from the three early flowering lines flowered significantly earlier compared to control lines (Table 3). The percentage of early flowering individuals in the early flowering epimutant lines (54.4%) was nearly three times the percentage of control lines (20.0%, Table 4) and a significant difference was detected between these three early flowering families and control lines (*p* = 0.01).

In order to further test the stability of the early flowering trait transmission in selected lines through both meiosis and mitosis, three extremely early flowering individuals EF4-9-13-15-18, EF4-14-7-6-12, and EF4-14-7-14-12 from the S4 families EF4-9-13-15, EF4-14-7-6, and EF4-14-7-14 were used to produce the fifth generation S5 families through seed reproduction. Mitotic heritability was evaluated in clonal daughter plant families EF4-9-13-15′, EF4-14-7-6′, EF4-14-7-14′, and control' generated from each of the S4 mother plants EF4-9-13-15, EF4-14-7-6, EF4-14-7-14. A highly significant difference was detected between the three early flowering families and control (*p* < 0.001) in the S5 generation, with 50.0% of the lines in these three families expressing the early flowering trait (Table 4). All three families (through both meiosis and mitosis) had significantly earlier flowering time compared to control (*p* < 0.05; Table 7). In the clonally propagated generation through mitosis, flowering time in early flowering families was around 6–10 days earlier than in control lines. These vegetatively propagated early flowering plants were on average advanced 5 days earlier than the lines which were propagated through seed.

**Table 7:** **Flowering time in S5 in early flowering lines through meiosis and mitosis**.

**Meiosis**				**Mitosis**			
**Families**	**Size**	**Mean**	***SD***	**Families**	**Size**	**Mean**	***SD***
EF4-9-13-15-18	12	75.9^*^	2.6	EF4-9-13-15′	15	17.3^***^	4.0
EF4-14-7-6-12	12	76.2^*^	2.4	EF4-14-7-6′	15	20.9^*^	4.4
EF4-14-7-14-12	12	74.3^***^	2.3	EF4-14-7-14′	15	21.3^*^	5.2
Control	24	78.5	2.8	Control′	15	27.1	7.1

* *p < 0.05*,

*** *p < 0.001 “Size” = the number of individuals*.

### Germination time in early flowering and control lines

A germination test was conducted in early flowering and control lines in order to determine if the early flowering lines were based on advanced plant development due to earlier germination. In total, five generations of early flowering lines derived from the EF4 line and eight control lines were evaluated for germination time, with each line consisting of 10–20 seeds. The average day to germination (DTG) of early flowering lines in 98 individuals was 12.6 with *SD* 2.6, which was similar to the average and *SD* of DTG (13.3 and 3.2 respectively) observed in the control lines (Supplementary File). No significant difference was found (*p* = 0.151) between the germination time of the early flowering and control lines in these two groups. Thus, the early flowering trait does not appear to be caused by advanced germination.

### DNA methylation profiles in early flowering lines and control lines using standard MSAP and novel MSAP

**Figure 6:**
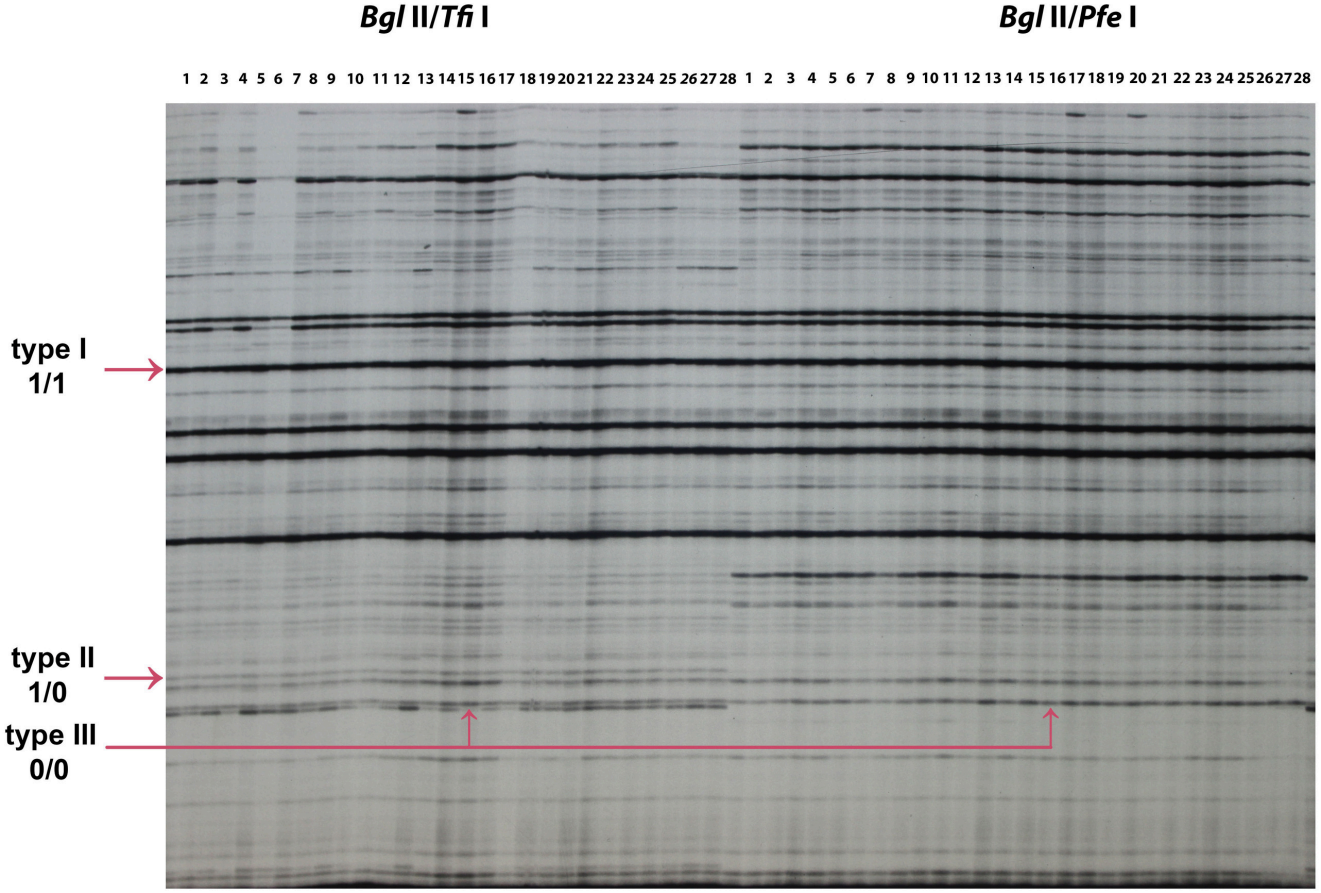
**MSAP profiles generated using new pairs of isoschizomeric combinations *(Bgl* II/*Tfi* I) and (*Bgl* II/*Pfe* I)**. 1–28: 28 epimutant lines used to test each isochizomeric combination. The arrows indicate type I (1/1), type II (1/0), and type III (0/0) bands amplified. The “1” represents the presence of bands and “0” represents the absence of bands, for example, type I (1/1) indicates the presence of a band in both *(Bgl* II/*Tfi* I) and (*Bgl* II/*Pfe* I), type II (1/0) indicates the presence of a band in *(Bgl* II/*Tfi* I) and the absence of a band in (*Bgl* II/*Pfe* I). The epimutant line 16 is used to illustrate type III (0/0) band.

A total of 166 MSAP loci were amplified and scored using six primer pairs with isoschizomer combinations *Bgl* II/*Tfi* I and *Bgl* II/*Pfe* I (Table 8). In order to compare the result of the standard MSAP, four primer pair combinations (Table 1) were used in isoschizomeric combinations *Eco*R I/*Hpa* II and *Eco*R I/*Msp* I in the same EF lines 1, EF lines 2, and control H4S8 lines. A total of 142 loci were amplified and scored in this standard MSAP approach. In total, three types of bands were grouped together based on scoring from these two MSAP approaches. There are two cytosines in double-stranded *Tfi* I/*Pfe* I recognition site GAWTC, resulting in three types of bands: type I representing non-methylation, type II representing hemimethylation, and type III representing full methylation (Figure 6). By contrast, there are more cytosine methylation patterns of double-stranded *Hpa* II/*Msp* I recognition site 5′-CCGG-3′ as demonstrated in the standard MSAP (Salmon et al., 2008; Xu et al., 2016). For the purpose of comparison, we combined both type III and type IV bands in *Hpa* II/*Msp* I as type III bands which represent both stranded cytosine methylation. Among the three groups studied (Table 8), the novel MSAP generated a higher percentage of non-methylated bands (around 80%) compared to the standard MSAP (nearly 60%). This can be explained by the different cytosine numbers in the two isoschizomer recognition sequences. There are two cytosines; one is in a CG methylation context in isoschizomers *Hpa* II/*Msp* I recognition site 5′-CCGG-3′, while *Tfi* I/*Pfe* I recognition site 5′-GAWTC-3′ only has one cytosine. Significantly reduced number of full-methylated bands (type III) was detected in EF lines 1 and EF lines 2 when compared to control using the novel MSAP method that exploited the *Tfi* I/*Pfe* I combination. In addition, in both novel and standard MSAP assays across all three groups, type I bands had the highest level, followed by type III bands, with type II bands at the lowest level.

**Table 8:** **The summary of three types of bands in control lines and two early flowering lines (EF lines 1 and EF lines2)**.

	**H4S8**		**EF lines 1**		**EF lines 2**	
	***Hpa* II/*Msp* I**	***Tfi* I/*Pfe* I**	***Hpa* II/*Msp* I**	***Tfi* I/*Pfe* I**	***Hpa* II/*Msp* I**	***Tfi* I/*Pfe* I**
Type I	83 (58.5%)	132 (79.5%)	82 (57.7%)	133 (79.9%)	81 (57.4%)	134 (80.8%)
Type II	15 (10.5%)	8 (4.8%)	14 (9.8%)	9 (5.8%)	14 (9.8%)	10 (5.9%)
Type III	44 (31.0%)	26 (15.7%)	46 (32.5%)	24 (14.3%)^*^	47 (32.8%)	22 (13.3%)^*^
Total	142	166	142	166	142	166

*Parentheses data represent the percentage when compared to the total number of bands*.

* *p < 0.05*.

**Figure 7:**
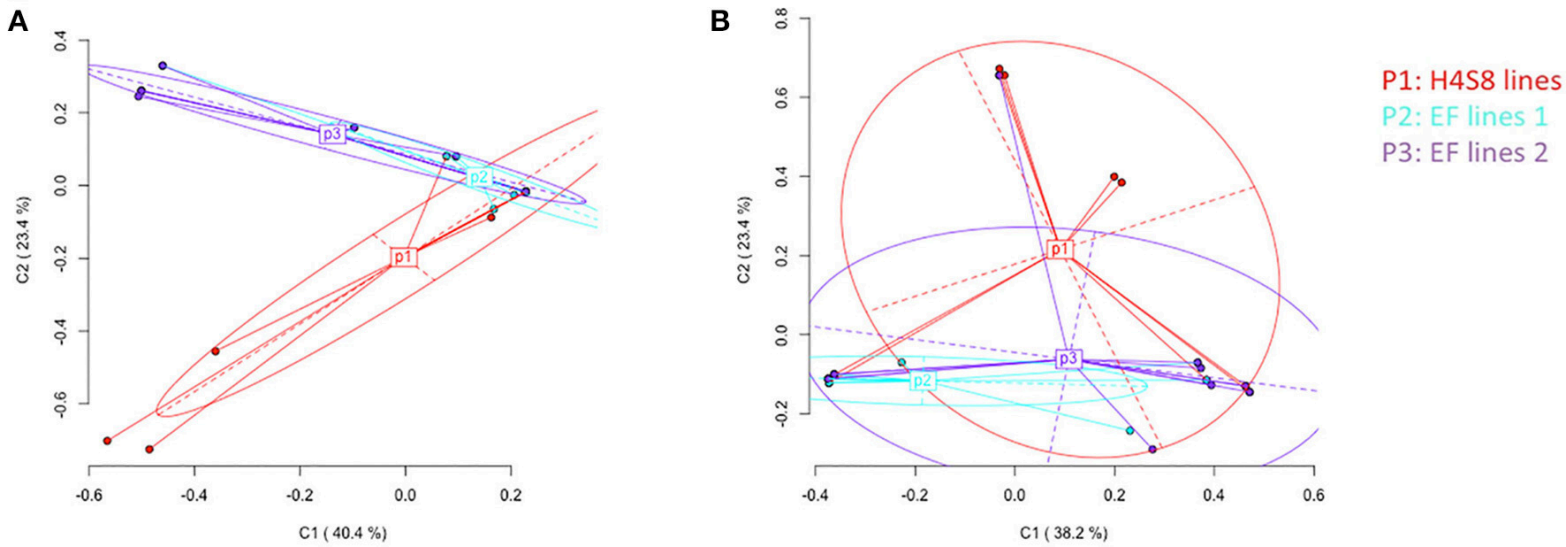
**Principal Coordinates Analysis (PCoA) for DNA methylation differentiation between control lines and 5-azaC treatment lines using polymorphic methylation-susceptible loci (MSL) data**. The percentages in the first two coordinates (C1 and C2) indicate the amount of variance contributed by them (shown in brackets). Color-labeled P1, P2, P3 are the centroids of corresponding group. P1 represents control H4S8 lines, P2 represents early flowering lines 1, P3 represents early flowering lines 2. **(A)** Data obtained from standard MSAP **(B)** data obtained from novel MSAP.

Epigenetic differentiation among control line H4S8, EF lines 1 and EF lines 2 based on all the loci was distinguished according to Principal Coordinate Analysis (PCoA). In the standard MSAP, a total of 142 loci were partitioned into 53 methylation-susceptible loci (MSL; 37%) and 89 non-methylated loci (63%). Only the 10 (19% of all MSL) polymorphic methylation-susceptible loci were used in the PCoA. By contrast, in the novel MSAP, a total of 166 loci were separated into 29 methylation-susceptible loci (17%) and 137 loci without methylation (83%). The number of polymorphic methylation-susceptible loci was 8 (28% of all MSL) and these loci were used in the PCoA. In the PCoA of H4S8 lines (P1), EF lines 1 (P2), and EF lines 2 (P3) using the standard MSAP, the first two coordinates explained 63.8% of the variance (Figure 7A). The P1 represented control H4S8 lines and was evenly spread out along the two coordinates. This indicated a similar pattern as reported in previous study (Xu et al., 2016). By contrast, most of the EF lines 1 (P2), EF lines 2 (P3) were distributed along the first coordinate. The significant difference in epigenetic variation among H4S8 lines (P1), EF lines 1 (P2), and EF lines 2 (P3) was detected (AMOVA, *P* = 0.0035). Pairwise PhiST comparisons indicated H4S8 lines (P1) were significantly different from EF lines 2 (P3) (*P* = 0.0166). Similar to the distribution of H4S8 lines (P1) of the standard MSAP, in the novel MSAP, the H4S8 control was also evenly distributed along the first two coordinates that accounted for 61.6% of the variance, and most EF lines 1 (P2) and EF lines 2 (P3) were distributed along the first coordinate (Figure 7B). A significant difference among the three groups was also observed (AMOVA, *P* = 0.0125). Further Pairwise PhiST comparisons indicated a significant difference between H4S8 lines (P1) and EF lines 1 (P2) (*P* = 0.0125). However, this contrasted with the standard MSAP experiment which detected significant differences between H4S8 lines (P1) and EF lines 2 (P3). Based on the significant difference of DNA methylation variation among P1, P2, and P3 groups in standard and novel MSAP, it appears that DNA methylation in 5′-CCGG-3′ (CG and CHG) accounted for the epigenetic variation in EF lines 2 (P3) while the DNA methylation in 5-GAWTC-3 (CG, CHG, and CHH) accounted for the epigenetic variation in EF lines 1 (P2).

## Discussion

In this study, an induced hypomethylated epi-mutant population of genetically identical individuals, through repeated selection of extreme phenotypic traits (such as early flowering) was shown to be heritable through meiosis over five generations (EF4). The epialleles induced by both genetic elements and environmental factors causing heritable traits is generally more difficult to characterize compared to alleles caused by DNA mutations (Paszkowski and Grossniklaus, 2011; Becker and Weigel, 2012). By growing the genetically identical plants under stable greenhouse environmental conditions and including controls in each generation, we aimed to address some of these issues. The generation of a hypomethylated population of *F. vesca* and the demonstration that alterations in DNA methylation patterns can expand phenotypic variance of quantitative characters in genetically uniform individuals provides a resource to test if DNA methylation polymorphisms can behave as epialleles that are heritable and subject to selection.

That selection of a phenotype based on epialleles is not always stable is generally known and has been reported (Hirsch et al., 2012). However, the plasticity of phenotypic traits has also been shown to be subject to selection (Scheiner, 1993; Van Kleunen and Fischer, 2003; Callahan and Pigliucci, 2005) and has not been widely investigated in horticulture crops. Therefore, the selection of epigenetic variants was conducted in flowering time and stolon emergence time in *F. vesca* in our research. In our study, 5-azaC was used as an epimutagen to induce the S0 hypomethylated population. This tool enables breeders to potentially increase selectable and heritable phenotypic variants of interest. In our selection from the S1 generation, we considered the extreme individuals rather than the families, which is different from the selection method used in classical breeding methods. In another study, selection was also made not only based on the mean but considered the variation and the extreme lines (Zhang et al., 2013). Although the initial production of the epimutants differed, the subsequent selection method of variants in our study was similar to the artificial selection for respiration and EUE reported in canola (*B. napus*; Hauben et al., 2009). The starting material in that study was selected based on plants with the highest and lowest respiration rate. Four lower respiration lines and three higher respiration lines were obtained after four rounds of selection of high and low respiration lines. This strategy was used in our work because the segregation of epialleles in the variants provided extremely early or late flowering time lines, or late stolon emergence lines without significant changes in the means due to the offset of two extremes compared to the control population. Therefore, only considering the family means might run the risk of omitting these variant phenotypes.

However, in the following S2 generation selection, only the families with statistically distinct phenotypic traits were considered and extreme individuals from these potential families were selected to continue the propagation through meiosis. This is because at this stage, the related epialleles across the genome is expected to be more stable and the individuals with potential epialleles are expected to show consistent phenotypic traits. All the early flowering and late stolon lines selected in the S2 generations revealed stable transmission of epialleles across one generation (stolon, LS-56) or three consecutive generations (early flowering, EF4-9 and EF4-14). However, the selected seven latest flowering individuals from the S1 generation failed to transmit to the next generation. This suggests the epialleles related to late flowering were reset through meiosis to cause a transient phenotypic change, as research indicated reversion of DNA methylation patterns affect the ability of epialleles to be subject to selection (Becker et al., 2011; Hirsch et al., 2012). Alternatively, no epialleles were present and the late flowering phenotype was induced by the toxic effect of 5-azaC or stress which disturbed the flowering time in the S0 generation (Jordan et al., 2015). In the S3, S4, and S5 generations, the early flowering trait was stably inherited. Thus, the early flowering related heritable epigenetic components may be stabilized under several rounds of phenotypic selection.

Several previous studies reported the inheritance of flowering time using epiRIL (Johannes et al., 2009; Roux et al., 2011; Zhang et al., 2013). This phenotypic inheritance of flowering time was correlated with the underlying DNA methylation alterations (Johannes et al., 2009). In our study, the early flowering line EF4, derived from 5-azaC treated genetically identical seeds, possessed the lowest methylation level detected by MSAP among all of the 22 epimutants selected to study, and the sequencing result verified no SNP observed in genes controlling flowering time in this line (Xu et al., 2016). To the best of our knowledge, there have been no studies of this nature in *Fragaria* or other members of the Rosaceae family. In a canola EUE study, the AFLP-test did not suggest any genetic polymorphism while MSAP revealed global hypomethylation and well-maintained differentially methylated fragments in selected lines (Hauben et al., 2009).

Through the germination time test, we verified a similar germination time between early flowering lines and control lines. This indicated the early flowering phenotype was not related to advanced development through earlier germination of the early flowering lines. In order to study the underlying mechanism of the early flowering trait, the MSAP method was used to screen the DNA methylation patterns of two early flowering lines EF line 1 and EF line 2. MSAP (standard) can determine the methylation at CG and CHG sites since the recognition sites are 5′-CCGG-3′ (Reyna-López et al., 1997). Since DNA methylation in plants occurs at three cytosine contexts: CG, CHG, and CHH, and different DNA methyltransferases that are involved in the *de novo* methylation and maintenance of DNA methylation of these contexts are related to particular biological functions (Teixeira and Colot, 2010; Kawashima and Berger, 2014), it is important to detect the DNA methylation profiles at both symmetric CG and CHG sites as well as the asymmetric CHH site. Therefore in this study, isoschizomers that can recognize the asymmetric CHH site were used in order to detect the cytosine methylation occurring at the asymmetric site, and further explored the maintenance of asymmetric cytosine methylation when the early flowering trait is passed to the following generations through meiosis. The isoschizomers *Tfi I* and *Pfe I* were used to study the epigenetic variation in two early flowering lines. To our knowledge, this is the first attempt to reveal the polymorphisms of cytosine methylation based on novel isoschizomers *Tfi I* and *Pfe I*.

In maize, the majority of meiotically heritable methylated cytosines occur at symmetric CG methylation context (Lauria et al., 2014). Similarly, a study in *Nicotiana tabacum* found symmetric CG methylation was stably inherited for at least two generations while methylation at CHG, CHH was not maintained (Dalakouras et al., 2012). This appeared to be associated with symmetric established cytosine methylation maintained through MET1 and CMT3 during each cycle of DNA replication. By contrast, methylation of CHH is not propagated based on template maintenance due to the asymmetrical sequence of CHH (Kankel et al., 2003; Henderson and Jacobsen, 2007). Thus, *de novo* methylation is required with the direction of small interfering RNA (Cao and Jacobsen, 2002). The material used in this study was derived from treating *F. vesca* seeds with the DNA demethylating reagent 5-azaC. The 5-azaC reagent is an analog of cytidine resulting in hypomethylation without specific preference, targeting both symmetric and asymmetric cytosines (Jones et al., 1983). Therefore, after the 5-azaC demethylation of symmetric CG and CHG methylation, the demethylated status of cytosine can be maintained through cell division across generations. In contrast, if 5-azaC demethylates CHH asymmetric sites, this demethylation can be transmitted via cell reproduction but will be reverted into the original state (methylated CHH) through *de novo* methylation after removal of 5-azaC. Accordingly, the clustering in P2 and P3 in standard MSAP may indicate symmetric CG, CHG methylation was maintained over generations while the reduced clustering of P2 and P3 according to the novel MSAP suggests the asymmetric CHH methylation pattern was not maintained over generations.

## Conclusion

Early flowering time is an important characteristic in strawberry breeding and production. From an evolutionary perspective, flowering time genes are often targets of selection which can be fixed to increase fitness in order to better adapt to the environment (Roux et al., 2006). For the first time, our research verified the inheritance of early flowering through both meiosis and mitosis in strawberry. However, the transmission of the late flowering phenotype was not observed in the S2 generation. Here the variants possessing early flowering phenotypes initially identified from a 5-azaC induced hypomethylated population of *F. vesca* were successfully inherited through five generations. This opens the door to a new approach of utilizing epigenetic variation as a breeding tool in horticulture crops for inheritance through either generative or vegetative propagation methods. Combined with the result of the standard MSAP method, the symmetric CG, CHG methylation, but not the asymmetric CHH, was found to be maintained over generations in the early flowering lines. The methylation maintenance at CG and CHG indicated the heritable alterations in DNA methylation at these sites and may be correlated with the early flowering phenotype. Using the epigenetic approach independent of DNA primary sequence changes is a potentially powerful new method to improve complex traits in plant development and breeding.

## Author contributions

JX performed the research and analyzed the data. JX drafted the manuscript. KT and SR edited the manuscript. JX, KT and SR conceived the idea and designed the research. All authors read and approved the final manuscript.

### Conflict of interest statement

The authors declare that the research was conducted in the absence of any commercial or financial relationships that could be construed as a potential conflict of interest.
