# Supplementary material for: Stable Epigenetic Variants Selected from an Induced Hypomethylated Fragaria vesca Population
Source: Front Plant Sci. 2016 Nov 29;7:1768. doi: 10.3389/fpls.2016.01768 (PMC5126047; doi:10.3389/fpls.2016.01768)
Supplement: Supplementary file 1 [file Presentation1.pdf]

Supplementary file

Germination time in early flowering lines and control lines.

	Generation	Families	Mean (day)	SD	Size
Eearly	S1	EF4	11.9	1.6	20
	S2	EF4-9	12.3	2.8	15
	S3	EF4-9-13	11.8	3.8	13
	S3	EF4-14-7	11.9	1.9	16
	S4	EF4-14-7-14	11.3	1.9	16
	S5	EF4-9-13-15-18	15.6	3.0	12
	S5	EF4-14-7-14-12	16.8	3.4	6
Control		Control 1	14.9	1.7	9
		Control 2	14.7	4.1	9
		Control 3	12.1	3.2	19
		Control 4	10.1	1.7	18
		Control 5	13.8	5.0	10
		Control 6	14.3	2.9	14
		Control 7	15.5	3.8	14
		Control 8	13.4	3.4	19

“Size” = the number of individuals.
